# Comparison of Artificial Neural Network and Multiple Linear Regression to Predict Cadmium Concentration in Rice: A Field Study in Guangxi, China

**DOI:** 10.3390/toxics13080645

**Published:** 2025-07-30

**Authors:** Junyang Zhao, Fuhai Zheng, Baoshan Yu, Guanchun Qin, Shunpiao Meng, Yuhang Qiu, Bing He

**Affiliations:** 1 Guangxi Key Laboratory of Agro-Environment and Agric-Products Safety, College of Agriculture, Guangxi University, Nanning 530004, China; zjy761174981@hotmail.com (J.Z.); a619650974@outlook.com (B.Y.); a2487960538@outlook.com (G.Q.); a2422450832@outlook.com (S.M.); a2470701350@hotmail.com (Y.Q.); 2 Agricultural Resources and Environmental Research Institute, Guangxi Academy of Agricultural Sciences/Guangxi Key Laboratory of Arable Land Conservation, Nanning 530004, China; fuhaizheng110@126.com

**Keywords:** prediction model, cadmium, soil-rice system, BP neural network

## Abstract

The translocation of cadmium (Cd) in the soil-rice system is complicated; therefore, most of the soil-plant models of Cd have not been extensively studied. Hence, we studied the back-propagation artificial neural network model (BP-ANN) and multiple regression model (MLR) to predict the cadmium (Cd) content in rice grain and soil through testing soil parameters. In this study, 486 pairs of rice grains and corresponding soil samples of 456 vectors were used for training + validation, and 30 vectors were collected from the southwestern karst area of Guangxi Province as a test data set. In this study, the Cd content in rice was successfully predicted by using the factors soil available cadmium (A_Cd_), total soil cadmium (T_Cd_), soil organic matter (SOM), and pH, which have a more significant impact on rice, as the main prediction variables. Root mean square error (RMSE), Relative Percent Difference (RPD), and correlation coefficient (R^2^) were used to assess the models. The R^2^, RPD, and RMSE values for R_Cd_ medium obtained by the MLR model with pH, T_Cd_, and A_Cd_ as entered variables were 0.551, 2.398, and 0.049, respectively. The R^2^ and RMSE values for R_Cd_ medium obtained by the BP-ANN model with pH, T_Cd_, and A_Cd_ as entered variables were 0.6846, 2.778, and 0.104, respectively. Therefore, it was concluded that BP-ANN was useful in predicting R_Cd_ and had better performance than MLR.

## 1. Introduction

Cadmium (Cd) is one of the most toxic heavy metals for both plant growth and human health. Cd is highly mobile, non-degradable, tends to persist in soil solution, and is easily taken up by plants, especially in food crops, and also induces adverse effects such as slow and stunted growth, and thus reduces the yield [1]. The uptake of Cd from the consumption of food has been a primary source of human exposure. Further, Cd exposure may cause many chronic health disorders, including gastrointestinal cancer, nephrotoxicity, and pulmonary diseases [2]. In China, about 2.786 × 10^9^ m^2^ of agricultural soils were polluted with Cd due to human activities such as wastewater irrigation, application of agrochemicals and manure, fossil fuel combustion, atmospheric deposition, mining, and metal processing [3,4].

Rice consumption is a very prominent route of heavy metal exposure to humans in areas where rice is a staple food source. Therefore, a thorough understanding of the factors influencing the concentration of Cd absorbed by rice and the relationship models between soil properties and rice Cd content (R_Cd_) has to be established to estimate the impact of rice consumption on human Cd burden. Soil pH was found to play the most critical role in determining the absorption, solubility, mobility, and eventual bio-availability of Cd in soil, while soil pH and R_Cd_ showed a negative correlation [5]. In addition, SOM can reduce the phyto-availability of Cd in soil through adsorption or forming stable complexes with humic substances [6]. While the changes in soil pH and dissolved organic carbon concentration could be critical for the increase in the availability of Cd in soils and Cd uptake [7]. In addition, R_Cd_ is also related to other soil properties, including clay content, cation exchange capacity (CEC), and other heavy metal concentrations, which also leads to a nonlinear relationship between R_Cd_ and soil properties [8]. Currently, many studies predict the Cd content in rice by constructing soil-rice coupling relationship models [9,10]. Compared to correlation analysis of a single variable, model analysis integrating multiple variables can predict the Cd content in rice more accurately, which is more conducive to assessing the harm of consuming rice from this region to the human body.

In the last few decades, several correlation models have been used to predict R_Cd_, such as multiple linear regression models (MLR) and artificial neural network models (ANNs) [9,10,11]. MLR is a kind of model that uses two or more explanatory variables to explain the dependent variables, as well as to represent the correlations between a number of inputs and a response of interest [12]. In addition, it is used to provide a large amount of information from a limited number of experiments by considering one variable at a time [13]. Rőmkens [14] measured Cd levels in brown rice that were predicted well (R^2^ > 0.8) based on Cd and Zinc in a 0.01 M CaCl_2_ extract or a soil–plant transfer model using the reactive T_Cd_, pH, and cation exchange capacity by MLR models. Brus [15] predicted R_Cd_ using the MLR model (R^2^ = 66.1%) with log (HNO_3_–Cd), pH, log (clay), and log (SOM) as predictors. Ding [16] used MLR analysis to predict Cd content of carrot (*Daucus carota* L.) with soil properties and found that T_Cd_, pH, and organic carbon were the significant variables contributing to the Cd concentration in carrot. Chen et al. [17] used MLR models to predict R_Cd_ and found that soil pH and SOM were the major factors influencing metal translocation from soil to rice. However, MLR cannot describe the nonlinear complex relationship between rice Cd and related parameters. Secondly, the disadvantage of the regression technique is that the different portions of the database may have different relationships between the soil properties and predictors. Thirdly, the regression equations (e.g., linear, logarithmic, or exponential) and predictors need to be determined as a priority [18].

An artificial neural network (ANN) is a series of mathematical algorithms that endeavors to identify complex non-linear relationships between input and output datasets, which is patterned after the biological nervous system [19]. Unlike the regression technique, ANN does not require any pre-defined model concept and can reliably recognize patterns from noisy and complex data and hence estimate their non-linear relationships [18]. ANNs have been used to predict the uptake of heavy metals in plants and persistent organic pollutants. Hou et al. [20] used BP-ANN optimized by the genetic algorithm to predict R_Cd_ based on soil properties. Wu et al. [21] utilized the Bayes classification statistical method and established a risk forewarning model for rice grain Cd pollution. Khazaei et al. [22] had chosen four-layer back-propagation networks with two hidden layers for prediction of crop yield. Jin [23] developed an improved genetic algorithm and a back-propagation neural network model for surface water quality prediction in the Ashi River, China, and the model had great performance both in prediction accuracy and reliability and effectively provided real-time early warning for emergency response.

MLR models do not meet the requirements of complex and nonlinear simulations and may fail under diverse conditions. Therefore, accurate and rapid detection of Cd concentration in rice grown in agricultural soil over large areas remains challenging. Moreover, most previous studies have primarily relied on soil-rice samples from specific regions, resulting in limited scope and scale, and hence exhibiting poor applicability to diverse regions [24]. The BP neural network is one of the most extensively used ANN models, consisting of a multi-layer network that uses a gradient descent-based algorithm for weight training [25]. Due to its strong fitting ability, the BP neural network is suitable for application of internal complex mechanisms such as the migration of heavy metals [20]. Therefore, in this study, we used the BP-ANN to predict R_Cd_ based on the influencing factors selected by correlation analysis. The specific objectives of this study include (1) using the basic soil property parameters (pH, SOM, A_Cd_, and T_Cd_) to accurately and rapidly predict the Cd content in rice and (2) comparing with the MLR model, verifying the feasibility of BP-ANN in predicting R_Cd_ in Northwest Guangxi.

## 2. Materials and Methods

### 2.1. Study Area

**Figure 1:**
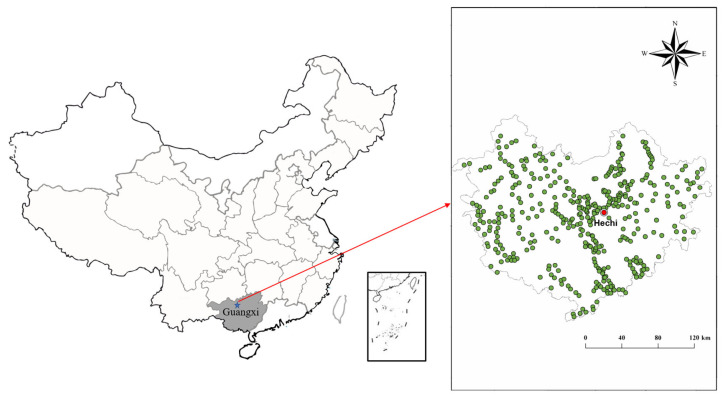
Sampling site and lithology sketch map of the study area.

The study zone (106.57°–109.15° E, 23.68°–25.62° N) is located in the southwestern karst area of Guangxi Province, China (Figure 1). Over 80% of the excess in heavy metals in the karst area was caused by regional geological background and soil weathering [26]. In addition, mining and irrigation by polluted river water or groundwater are other important factors leading to excessive heavy metals in cultivated soil.

### 2.2. Sampling and Pre-Treatment

During 2015 at harvest, a total of 486 paired samples of topsoil (0–20 cm in depth) and rice grain were collected at fixed points following a five-point mixing sampling method in the research area. Soil samples were air-dried and sieved through a 2 mm polyethylene sieve for measurement of soil pH. A portion of these samples were ground and sieved through a 0.149 mm (100 mesh) for T_Cd_, A_Cd_, and SOM analyses. Rice grains were washed with tap water and then three times with deionized water and oven-dried at 45 °C to a constant weight. After removal of the hulls, oven-dried rice grains have been milled to <200 μm for measurement of Cd content.

### 2.3. Soil and Rice Sampling

The physical and chemical properties of soil were determined according to the method described in the Analysis Methods of Soil Agricultural Chemistry [27]. The pH was measured by the potentiometric method, and the water:soil ratio was 2.5:1.0; the SOM content was measured by the potassium dichromate external heating method; the T_Cd_ content in soil was determined by 2:2:1 HNO_3_:HClO_4_:HF (v:v:v) digestion, the A_Cd_ content was extracted by DTPA solution [28], and the Cd contents of the digestion solution and extract solution were determined by atomic absorption spectrophotometer (PinAAcle 900 T, PerkinElmer, Waltham, MA, USA). R_Cd_ was determined according to Lu et al. [29]. Brown rice was baked at 70 °C to constant weight, then pulverized and passed through a 100-mesh sieve. Nitric acid was used to carry out microwave digestion with a microwave digestion apparatus (MARSXpress, CEM, Matthews, NC, USA); the Cd content was measured using a graphite furnace atomic absorption spectrometer (PinAAcle900T, PerkinElmer, Waltham, MA, USA); and the quality control was carried out by using the GBW100348 [30] plant standard material of the National Institute of Metrology, China.

### 2.4. Statistical Analysis

The SPSS^®^ 10.0 (SPSS Inc., Chicago, IL, USA) software was used for data analysis. Correlation analysis was performed by using Pearson’s correlation coefficient test to determine the relations between different variables, while two-way analysis of variance (ANOVA) was conducted to identify the differences among groups, and relationships were considered significant at *p* < 0.05 and *p* < 0.01.

## 3. Development of Prediction Models

### 3.1. Data Pre-Processing Phase

To eliminate the impact of different platforms on the results of network training and improve the efficiency and quality of network training, this study normalized the rice Cd content and soil data to avoid significant differences between input and output data [31]. The sample data were normalized to [−1, 1] using the following formula:

X_norm_ = (a − b) × (x − x_min_)/(x_max_ − x_min_) + b (1)

where x, x_norm_, x_max_, and x_min_ are the actual value, normalized value, maximum value, and minimum value of the sample, respectively, and a and b are the maximum and minimum values of the normalized interval, respectively.

### 3.2. Development of BP-ANN Model

**Figure 2:**
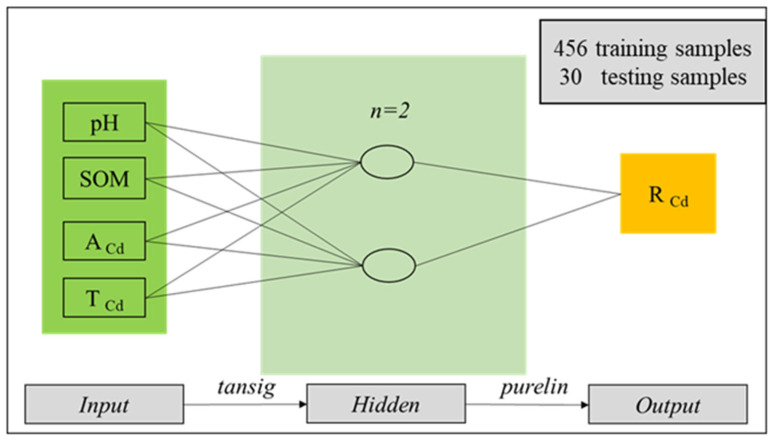
General flow-chart of BP-ANN model to predict Cd in rice.

In order to accurately and rapidly predict Cd content in rice by using basic soil property parameters. This study uses MATLAB (2015) to build a neural network model, with SOM, pH, A_Cd_, and T_Cd_ as input parameters and R_Cd_ as output parameters (Figure 2). These indicators will be measured, and a database will be established during the national soil survey. Our model will also be submitted to the government, which will extract data from the database to use this model for predicting Cd content in rice and creating a predictive distribution map of rice quality and safety in Guangxi. The structure of the BP-ANN consists of an input layer, a hidden layer, and an output layer, which includes multiple neurons, and each layer is linked by connection weights. The number of neurons in the input and output layers depends on the input and output variables, respectively. One (or more) hidden-neuron layer receives weight from each input neuron and adds them up to a weight value and then passes the results through a non-linear function [32].

Selecting the number of neurons in the hidden layers is a very crucial part of deciding the overall neural network architecture because the number of hidden layers and the number of neurons in each hidden layer may affect the training efficiency and the precision of prediction [18]. In most cases, BP-ANN with a single hidden layer is sufficient to provide an accurate approximation and useful for limiting the calculation [31]. The optimal number of neurons in the hidden layer was determined by the trial and error method to minimize the prediction error. The number of neurons was chosen in the hidden layer by the following empirical Formula (2):

(2) $$n\;=\;\sqrt{p+q}+a$$

where n is the number of hidden layer nodes, *p* is the number of input layer nodes, *q* is the number of output layer nodes, and a is an integer between 1 and 10. In this study, the numbers of neurons 2, 3, 4, 5, and 6 were selected for modeling.

In the ANN models, an essential step is the splitting of the available data into three subsets: training, validation, and test data sets [33]. In this paper, the required data are sampled into two subsets with a training + validation set size of 456 vectors and a test set size of 30 vectors (randomly selected data) that were used to predict R_Cd_. The ratio of training set and validation set is 1:1 (231:225), 2:1 (306:150), and 3:1 (343:113), respectively. Meanwhile, the cross-validation technique is used during the training process to divide the training and validation data sets and prevent the training model from being overfitted. For the training of datasets, the trainlm (Levenberg-Marquardt backpropagation) function was used as a learning algorithm in the developed ANN model. Choose the tansig function for the connection between the input layer and the hidden layer, and opt for the purelin function for the connection between the hidden layer and the output layer.

### 3.3. Development of MLR Model

To assess and compare the performance of BP-ANN models, we used SPSS 21 to establish multiple linear regression models (MLR). MLR is a classic simple linear regression model from single to multiple predictors where the objective is to deduce a model that can exhibit the maximum deviations in the predictor data to evaluate their corresponding regression coefficients [34]. MLR is often used as a reference to evaluate other non-linear models. The logarithm of T_Cd_, A_Cd_, and R_Cd_ based on 10 is taken, respectively, and then a regression analysis model is carried out. The following regression prediction model was obtained:

    log R_Cd_ = a + b pH + c SOM + d log T_Cd_

    log R_Cd_ = a + b pH + c SOM + d log A_Cd_

log R_Cd_ = a + b pH + c log T_Cd_ (3)

### 3.4. Evaluation Criteria for Model Performance

In order to assess the performance and stability of the models, the difference between the predicted values and the experimental values was evaluated. The root mean square error (RMSE), coefficients of determination (R^2^), and the ratio of standard deviation (RPD) values were as follows [35,36]:

(4) $$SEP={\left[\frac{\sum_{i=1}^{n}{\left(Y_{i}^{p}-{\hat{Y}}_{i}^{p}\right)}^{2}}{n-1}\right]}^{1/2}$$

*SEP* is the Standard Error of Prediction, $Y_{i}^{p},{\hat{Y}}_{i}^{p}$ are the measured and predicted values of the predicted samples, and n is the number of modeling samples.

(5) $$RPD=\frac{S.D}{SEP}$$

*S.D.* is the measured standard deviation.

(6) $$RMSE=\sqrt{\frac{1}{n-k-1}\sum_{i=1}^{n}{\left(Y_{i}-Y_{i}^{J}\right)}^{2}}$$

The RMSE was used as a performance measure for the training performance using Equation (5). RMSE values close to zero also denote excellent performance on the part of the model, where *Y_i_* and *Y_i_^J^* are the measured and predicted values of the modeling samples, n is the number of modeling samples, and k is the number of factors contained in the model.

## 4. Results and Discussion

### 4.1. Changes in Soil Properties and Cd Concentration

**Figure 3:**
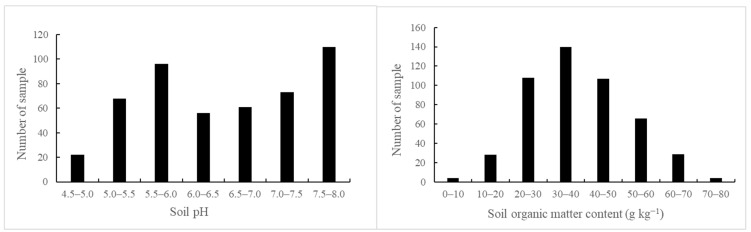
Variation in soil pH and soil organic matter content of different soil samples.

Data on soil properties and R_Cd_ are listed in Table 1. Soil pH ranged from 4.43 to 8.02. There are 242 samples having acidity values below 6.5, accounting for 50% of the total samples (Figure 3), and more differences in soil pH were due to the two main types of soil in the study area. One is red soil, with the characteristic of acidity to strong acidity, and the pH is usually in the range of 4–6. The other is limestone soil, usually 7.5–8.5 of alkaline pH. Further, SOM ranged from 4.07 g kg^−1^ to 75.43 g kg^−1^ with an average value of 38.44 g kg^−1^ (Figure 3). The content of organic matter was mainly in the range of 20–50 g kg^−1^, which belongs to the normal content range. Samples with SOM below 20 g kg^−1^ account for 6.6% of the total samples, indicating that the surveyed soil was fertile.

**Figure 4:**
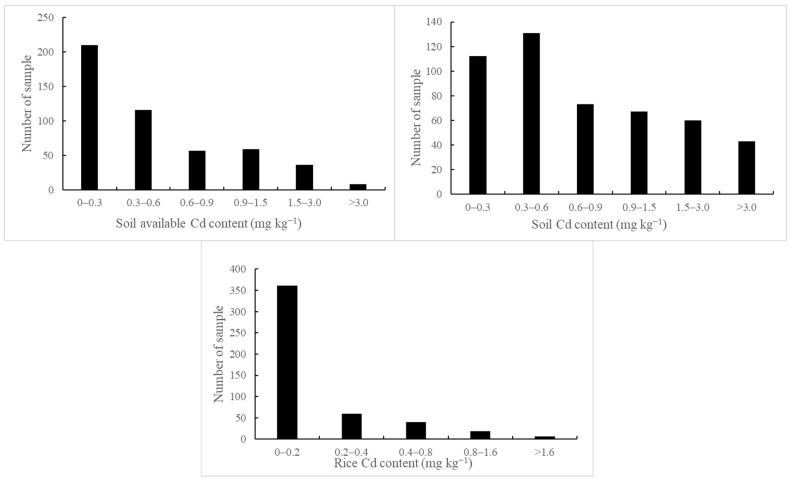
Variation in soil total and available Cd content and rice grain Cd content of different soil and plant samples.

T_Cd_ ranged from 0.093 to 8.76 mg kg^−1^ (Figure 4), and about 23% of the samples had T_Cd_ lower than 0.3 mg kg^−1^, which was lower than the risk screening values for the soil contamination of agricultural land. A_Cd_ ranged from 0.002 to 4.860 mg kg^−1^ with the mean value of 0.61 mg kg^−1^ (Table 1). A_Cd_ of the samples between 0 and 0.3 mg kg^−1^ was the most, accounting for 43% of the total sample size. High concentrations of A_Cd_ allow for permissive transfer from soil to rice grains and increased Cd accumulation in rice grains.

R_Cd_ was mainly distributed between 0.001 and 4.43 mg kg^−1^, with an average value of 0.2 mg/kg and a median value of 0.07 mg kg^−1^ (Table 1). About 74% of the rice samples had cadmium content between 0 and 2 mg kg^−1^ (Figure 4). According to the agricultural industry standard of the People’s Republic of China [37], in this study area, rice samples have 26% more of the exceeding Cd than the standard cadmium.

### 4.2. Effect of Soil Properties on R_Cd_

Pearson correlation analysis was performed to quantify the relationship between different parameters (Table 2). Correlation analysis showed that R_Cd_ was significantly positively correlated with T_Cd_ (0.124 **) and A_Cd_ (0.220 **) in soil and significantly negatively correlated with soil pH (−0.216 **), while negatively uncorrelated with SOM (−0.078). Chen considered SOM an important factor influencing the availability of heavy metals in soils [22], and we found that SOM was significantly correlated with A_Cd_ (Table 2).

Most studies showed that the increase in T_Cd_ and A_Cd_ led to the increase in R_Cd_ [38]. As shown in Table 1, T_Cd_ is an important factor in determining R_Cd_ (r = 0.124). In the previous study, A_Cd_ was positively correlated with T_Cd_, SOM, and pH [39]. It is commonly accepted that the A_Cd_ could be a better indicator of bio-availability and toxicity than the T_Cd_ [40], and the bio-availability of Cd was strongly correlated with the ability to transfer from soil into plants [41].

A significant positive correlation between A_Cd_ and pH of the soils was also observed (*p* < 0.05). pH is an important factor affecting R_Cd_ by determining the bio-availability of Cd in soil. Increasing soil pH could immobilize heavy metals by increasing soil adsorption and enhancing the easily bio-available forms to immobile forms [42]. The decreased soil pH induced heavy metal desorption from soil constituents, increased mobility and bio-availability of Cd, and increased Cd uptake by rice [12]. In our research, the correlation between A_Cd_ and pH was not obvious. The possible reason is that our soil samples were mainly composed of acid red soil and alkaline lime soil. pH was different between soils, which may affect the correlation analysis of A_Cd_ and pH.

These findings indicate that soil properties other than SOM, such as pH and T_Cd_, had a strong influence on the available Cd in soil and therefore the amount of Cd absorbed by rice grains. SOM may have different functions and generate noise to data. On the one hand, SOM can reduce the Cd availability in the soil through adsorption or forming stable complexes with humic substances [43]. On the other hand, SOM supplies organic chemicals, acting as chelates, to the soil solution and enhances Cd availability to rice [44]. The SOM appeared as an indirect variable affecting Cd uptake by rice; therefore, the SOM was also used as a predictor.

### 4.3. Development of BP-ANN Model for R_Cd_

The best architecture (based on prediction accuracy on the training and test sets) was a fully connected three-layer, feed-forward network with one hidden layer. These architectures were used for all the networks, and their prediction accuracy was assessed on the same set of validation cases. The current stage of this study aims to accurately and rapidly predict the Cd content in rice using BP-ANN model. We chose pH, SOM, A_Cd_, and T_Cd_ as the four input parameters because the government has established databases for these parameters. We aim to utilize the existing government databases to establish a prediction model. For the purpose of rapid prediction, we test the four parameters in groups: (1) pH, SOM, T_Cd_; (2) pH, SOM, A_Cd_; (3) pH, A_Cd_, T_Cd_. For each subset of selected variables, BP-ANN models were developed (Table 3). There was no significant correlation between SOM and R_Cd_, so there was no model test with parameters of SOM, A_Cd_, and T_Cd_. At the same time, the model with four input parameters (pH, SOM, A_Cd_, T_Cd_) is not established, because we have less data, the accuracy of the model may be reduced, and the increase in parameters will lead to the increase in the running time of the model, which cannot achieve the purpose of rapid prediction.

Through comparative analysis, when the number of neurons in the hidden layer is 2 and the ratio of training set to correction set is 2:1, the prediction accuracy of Mode I is the best, and the RPD value is 2.165, which is significantly higher than the RPD value under other conditions (Table 4). The best structure of Mode II is the same as Mode I, but the RPD value is higher, which is 2.488. In Mode III, the best architecture was a fully connected three-layer, one hidden layer with 3 hidden nodes; the ratio of training set to correction set is 2:1.

### 4.4. MLR for Predicting Cd Content in Rice

A best-fit model using MLR to predict R_Cd_ was developed. Table 5 presented the RPD values for three models. After comparing the RPD, the MLR of Mode II had the best prediction accuracy. The following model, including independent variables, was adopted:

log R_Cd_ = 0.851 − 0.226 pH − 0.007 SOM + 0.557 logA_Cd_

It was found that RPD values were above 2.342, which means that rice Cd content could be attributed to soil pH, OM, and T_Cd_ using MLR models, but the accuracy was lower than the best ANN model II (RPD 2.488) and the best ANN model III (RPD 2.422).

## 5. Comparison of Different Models

**Figure 5:**
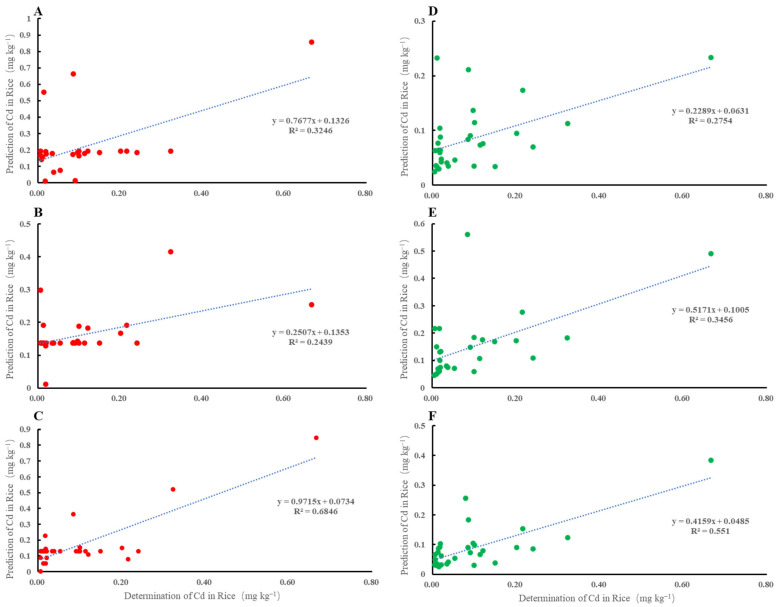
Comparison of the predicted values of the different BP-ANN and MLR models for the best input layer with the actual value ((**A**): Mode I, BP; (**B**): Mode II, BP; (**C**): Mode III, BP; (**D**): Mode I, MLR; (**E**): Mode II, MLR; (**F**): Mode III, MLR).

To compare the performance of the MLR and BP-ANN predictions, the training and testing results of these methods were shown in Table 6 and Figure 5, respectively. Comparing the results of these methods and their models showed that the performance values of BP-ANN were better than MLR. The RMSE values of BP-ANN were lower than those of MLR in Mode I and Mode II, but not in Mode III (Table 6). For all models, according to the derived results of the ANN method, based on the testing data set, the RPD ranged from 2.670 to 2.853, while the corresponding range of 2.398–2.581 was obtained based on MLR (Table 6). The predicted outputs (R_Cd_) of the testing datasets from both the well-trained BP-ANN model and the MLR model were compared with the actual values, as shown in Figure 5. The correlation will be better when the correlation coefficient is close to 1, namely, when the predicted value is closer to the observed value. It can be seen that the performance analysis of validation errors was similar to the performance analysis of test errors, indicating that the trained BP-ANN can generalize and the optimal network obtained in the model training process based on the training and validation data were therefore valid. A comparison of the performance analysis of test errors between BP-ANN and MLR shows the differences in prediction accuracy, which indicates that the BP-ANN model can effectively improve R_Cd_ prediction accuracy. It can be seen that the ANN predictions have a good relation with the experimental results, which means that the ANN has been sufficiently trained.

Table 6 showed that the BP-ANN model was superior to the MLR model in terms of predicting R_Cd_. Although the RMSE decreased from 0.104 in the BP-ANN model to 0.049 in the MLR model, the R^2^ increased from 0.551 in the MLR model to 0.6846 in the BP-ANN of Mode III (Figure 5C,F). Figure 5 shows the observed and predicted Cd values obtained through the BP-ANN model during the testing phase. The comparison results between Figure 5 indicated that the BP-ANN model outperformed the MLR model in the prediction of R_Cd_. The findings can be explained as follows: The correlation between rice Cd content and soil parameters tends to be nonlinear. The most significant problem in MLR is the assumption of a linear input-output relationship, but this assumption is unacceptable for complex systems. Conversely, a great advantage of ANNs is their ability to model non-linear relationships. There was no big difference between the goodness-of-fit measures for respective models developed by MLR (Figure 5D–F) methods.

Therefore, when compared with multiple regression analysis, the BP neural network can reveal the nonlinear relationship between Cd concentration in rice grain and soil properties better, which overcomes the shortcomings of simulation by the multiple regression model using complex factors. According to Maran [45], the BP-ANN model having the highest prediction performance among the models may be due to the tendency of ANNs to approximate the non-linearity of the system. Olawoyin [46] described results with the use of BP-ANN to model the relationship between soil input data and the content of soil carcinogenic PAHs, which performed better (R^2^ = 0.99) than conventional models such as the MLR. Keshavarzi and Sarmadian [47] developed the performance of the MLR and ANN models for predicting soil parameters using easily measurable characteristics of clay and organic carbon. Results showed that ANN with seven neurons in the hidden layer had better performance in predicting soil CEC than MLR. The accuracy of rice cadmium prediction in contaminating soils using machine learning models was increased compared with linear regression. The comparison between ANN and MLR models has shown that ANN models have better precision with a higher coefficient of correlation than MLR models [48].

Overall, the optimal model for estimating the Cd concentration of rice grain is the BP neural network model because the BP neural network model can be used as a black-box model to predict an individual variable through a complex interaction factor and to process complex and fuzzy mapping relationships without knowing the relationship between the distribution form and variables. Therefore, when compared with multiple regression analyses, the BP neural network can reveal the nonlinear relationship between Cd concentration in rice grain and soil properties better, which overcomes the shortcomings of simulation by the multiple regression model using complex factors.

Our models did not incorporate some parameters that likely influence the Cd content of rice, such as irrigation patterns, CEC, clay content, rice varieties, Fe and S concentration, microbes in soil, and temporal or spatial associations in the ANNs. Among them, rice varieties exhibit significant variations in their propensity to accumulate heavy metals. Incorporating the genes primarily responsible for Cd absorption and translocation in these rice varieties into the model would further refine the relevant indicators influencing Cd migration within the soil-rice system. However, a limited dataset may result in weaker model performance. Additionally, this study is confined to soil-rice samples from specific regions, thus limiting its scope. Therefore, while enhancing the relevant indicators of the model, it is imperative to broaden the research scope and scale and augment the dataset substantially, which will enhance the accuracy of the predictive model.

## 6. Conclusions

The migration of Cd in the soil-rice system is very complicated. The black box characteristics of BP-ANN enable it to predict the content of Cd in rice grains through relatively stable physical and chemical properties in soil. In this study, the Cd content in rice was successfully predicted by using the factors that have a more significant impact on R_Cd_ (such as A_Cd_, T_Cd_, SOM, and pH) as the prediction variable. ANN models gave a higher R^2^ and lower RMSE compared to the MLR and indicated that ANN was a more powerful tool than MLR. BP-ANN can be used as a fast and useful tool for predicting Cd concentration in soil, which can contribute to better assessments on the safe use of soil and the production of quality food. The research results can lay a theoretical foundation for the application of neural network technology in the field of crop heavy metal content prediction and provide a more accurate rice prediction method. It provides a theoretical reference for the government to make and implement relevant policies on the classification and control of heavy metal pollution in rice fields.

## Figures and Tables

**Table 1:** Soil properties and Cd in soil and rice in 2015.

Parameters	Mean	Minimum	Maximum
pH	6.14	4.56	8.00
SOM (g kg^−1^)	37.29	4.07	75.43
A_Cd_ (mg kg^−1^)	0.61	0.002	4.86
T_Cd_ (mg kg^−1^)	1.13	0.093	8.76
R_Cd_ (mg kg^−1^)	0.200	0.001	4.431

Note: A_Cd_ is the available Cd content in soil; T_Cd_ is the total Cd content in soil; R_Cd_ is the total Cd content in rice.

**Table 2:** Correlation analysis of R_Cd_ and soil parameters.

Indications	pH	SOM	A_Cd_	T_Cd_	R_Cd_
pH	1	0.123 **	0.112 *	0.232 **	0.216 **
SOM		1	0.256 **	0.286 **	−0.078
A_Cd_			1	0.827 **	0.220 **
T_Cd_				1	0.124 **
R_Cd_					1

Note: ** Significant correlation at 0.01 level (bilateral); * Significant correlation at 0.05 level (bilateral).

**Table 3:** Parameters of BP-ANN models.

Model	Input	Output
Model I	pH, SOM, T_Cd_	R_Cd_
Model II	pH, SOM, A_Cd_	R_Cd_
Model III	pH, T_Cd_, A_Cd_	R_Cd_

**Table 4:** BP-ANN model prediction results.

Implied Layer of Neurons	Training Set and Calibration Set Ratio	RPD		
		Model I	Model II	Model III
2	1:1	0.992	1.488	2.368
	2:1	2.165	2.488	2.036
	3:1	1.892	2.409	2.015
3	1:1	1.172	1.504	2.225
	2:1	1.683	2.315	2.422
	3:1	1.464	1.328	2.282
4	1:1	1.037	1.389	2.221
	2:1	1.165	1.246	1.944
	3:1	1.492	2.386	2.172
5	1:1	0.846	1.703	2.278
	2:1	1.040	1.578	2.185
	3:1	1.132	1.153	2.047
6	1:1	1.024	0.981	2.296
	2:1	0.901	1.312	2.081
	3:1	0.910	1.749	2.157

Note: Mode I: pH, SOM, and T_Cd_ as input parameters; Mode II: pH, SOM, and A_Cd_ as input parameters; Mode III: pH, T_Cd_, and A_Cd_ as input parameters.

**Table 5:** Regression equations for different models.

Model	Training and Calibration Set Ratio	MLR	RPD
Model I	1:1	log R_Cd_ = 0.517 − 0.23 pH + 0.483 logT_Cd_	2.032
	2:1	log R_Cd_ = 0.973 − 0.263 pH − 0.007 SOM + 0.615 logT_Cd_	2.226
	3:1	log R_Cd_ = 1.0115 − 0.265 pH − 0.008 SOM + 0.596 logT_Cd_	2.224
Model II	1:1	logR_Cd_ = 0.385 − 0.204 pH + 0.391 logA_Cd_	2.314
	2:1	log R_Cd_ = 0.851 − 0.226 pH − 0.007 SOM + 0.557 logA_Cd_	2.342
	3:1	log R_Cd_ = 0.814 − 0.221 pH − 0.008 SOM + 0.502 logA_Cd_	2.232
Model III	1:1	log R_Cd_ = 0.517 − 0.236 pH + 0.48 logT_Cd_	2.032
	2:1	log R_Cd_ = 0.645 − 0.258 pH + 0.533 logT_Cd_	2.172
	3:1	log R_Cd_ = 0.682 − 0.262 pH + 0.509 logT_Cd_	2.164

Note: Mode I: pH, SOM, and T_Cd_ as input parameters_;_ Mode II: pH, SOM, and A_Cd_ as input parameters; Mode III: pH, and T_Cd_ as input parameters.

**Table 6:** Forecast results for different models.

Model	Model Type	RPD	RMSE
Mode I	BP-ANN	2.670	0.119
	MLR	2.573	0.135
Mode II	BP-ANN	2.853	0.102
	MLR	2.581	0.123
Mode III	BP-ANN	2.778	0.104
	MLR	2.398	0.049

Note: Mode I: pH, SOM, T_Cd_; Mode II: pH, SOM, A_Cd_; Mode III: pH, ACd, T_Cd_.

## Data Availability

All data generated or analyzed during this study are included in this published article.
